# Social Withdrawal in Adolescence and Early Adulthood: Measurement Issues, Normative Development, and Distinct Trajectories

**DOI:** 10.1007/s10802-018-0497-4

**Published:** 2018-11-27

**Authors:** Stefania A. Barzeva, Wim H. J. Meeus, Albertine J. Oldehinkel

**Affiliations:** 1 Interdisciplinary Center Psychopathology and Emotion Regulation, University Medical Center Groningen, University of Groningen, Groningen, The Netherlands; 2 Research Center Adolescent Development, Utrecht University, Utrecht, The Netherlands

**Keywords:** Social withdrawal, Trajectories, Measurement invariance, Adolescence, Early adulthood

## Abstract

Social withdrawal during adolescence and early adulthood is particularly problematic due to the increasing importance of social interactions during these ages. Yet little is known about the changes, trajectories, or correlates of being withdrawn during this transition to adulthood. The purpose of this study was to examine the normative change and distinct trajectories of withdrawal in order to identify adolescents and early adults at greatest risk for maladjustment. Participants were from a Dutch population-based cohort study (Tracking Adolescents’ Individual Lives Survey), including 1917 adolescents who were assessed at four waves from the age of 16 to 25 years. Five items from the Youth Self Report and Adult Self Report were found to be measurement invariant and used to assess longitudinal changes in social withdrawal. Overall, participants followed a U-shaped trajectory of social withdrawal, where withdrawal decreased from ages 16 to 19 years, remained stable from 19 to 22 years, and increased from 22 to 25 years. Furthermore, three distinct trajectory classes of withdrawal emerged: a low-stable group (71.8%), a high-decreasing group (12.0%), and a low-curvilinear group (16.2%). The three classes differed on: shyness, social affiliation, reduced social contact, anxiety, and antisocial behaviors. The high-decreasing group endorsed the highest social maladjustment, followed by the low-curvilinear group, and the low-stable group was highly adjusted. We discuss the potential contribution of the changing social network in influencing withdrawal levels, the distinct characteristics of each trajectory group, and future directions in the study of social withdrawal in adolescence and early adulthood.

**Electronic supplementary material:**

The online version of this article (10.1007/s10802-018-0497-4) contains supplementary material, which is available to authorized users.

Social withdrawal is an umbrella term referring to an individual’s voluntary self-isolation from familiar and/or unfamiliar others through the consistent display of solitary behaviors (Rubin et al. 2009) such as shyness, spending excessive time alone, and avoiding peer interaction. Underlying motivations to withdrawal may vary between individuals (Asendorpf 1990; Ozdemir et al. 2015; Wang et al. 2013). Based on varying approach-avoidance motivations, Coplan and Armer (2007) identified three types of social withdrawal: shyness (high approach, high avoidance), unsociability (low approach, low avoidance), and social avoidance (low approach, high avoidance). Phenotypic withdrawal behaviors overlap across withdrawal types. In the current manuscript, we use the term ‘social withdrawal’ to refer to the global, multidimensional, behavioral phenotype of voluntary self-isolation. Socially withdrawn adolescents (ages 10–20) and early adults (ages 20–25) face challenges both parallel to those of withdrawn children and unique to the transition to adulthood (Hamer and Bruch 1997; Nelson et al. 2008; Nelemans et al. 2014; Rowsell and Coplan 2013). Yet only a small segment of the withdrawal literature has examined the normative changes, heterogeneous trajectories, or correlates of withdrawal during these ages. More research is needed to increase our understanding of the specific roles social withdrawal plays in the lives of adolescents and early adults in order to increase well-being during this transitional period and promote positive adjustment thereafter. The current study contributes to the literature by examining the developmental changes of social withdrawal while considering measurement issues pertinent to developmental research. The following sections review the current state of knowledge of both aspects.

## Normative Patterns and At-Risk Trajectories of Social Withdrawal

Socio-cognitive abilities allow youth to evaluate themselves in their social contexts. These abilities improve during early adolescence, which leads to growing attention to and perceived importance of adolescents´ social functioning relative to their peers, and a heightened sensitivity to how they are perceived by their peers (Gazelle and Rubin 2010; Steinberg and Morris 2001; Weems and Costa 2005; Westenberg et al. 2004). This increase of evaluative concerns might induce an increase in social withdrawal in early and middle adolescence. With greater social experience and brain maturation (Choudhury et al. 2006), and larger social networks (Wrzus et al. 2012), evaluative concerns likely diminish during late adolescence and early adulthood, thereby decreasing social withdrawal. To date, only two studies have reported on changes in withdrawal through adolescence and early adulthood, with contradicting findings. The first found a small increase in parent-reported social withdrawal from ages 4 to 18 years (Bongers et al. 2003), while the second found a small decrease in parent-reported shyness from ages 4 to 23 years (Dennissen et al. 2008). Neither study tested for potential curvilinear associations of withdrawal over time. Curvilinear associations are likely because several phenomena related to social evaluation and social withdrawal have been found to follow a curvilinear association with increases during early adolescence and decreases during late adolescence and adulthood, such as self-consciousness (Rankin et al. 2004), social conformity (Sistrunk et al. 1971), perceived importance of popularity (LaFontana and Cillessen 2010), and social anxiety (Nelemans et al. 2014).

Regardless of the mean-level trajectory of withdrawal, not all adolescents and adults will follow the general developmental pattern. On the individual level, increases, decreases, and stability in social withdrawal are all likely. The contradictory findings and weak effects in the two mean-level studies may point to heterogeneous patterns of withdrawal trajectories. In the preadolescent social withdrawal literature, distinct trajectories of increasing, decreasing, and low-stable withdrawal have been consistently reported using peer nominations of anxious withdrawal (Booth-LaForce et al. 2012; Oh et al. 2008) and teacher-reported social withdrawal (Booth-LaForce and Oxford 2008). In this body of work, the majority of children have very low and stable levels of withdrawal over time. The increasing withdrawal group exhibits the highest maladjustment, and the decreasing group exhibits intermediate maladjustment levels. Only Tang and colleagues (Tang et al. 2017) have examined the trajectories of withdrawal into adolescence and adulthood, with participants aged 8 to 35 years. They found the same three withdrawal trajectories as reported in the preadolescent literature and comparable group differences in maladjustment. However, it is too early for firm conclusions, because Tang’s study had three major limitations. First, curvilinear mean-level patterns and distinct curvilinear trajectories were not reported. Second, informant effects were introduced by assessing social withdrawal with parent-reports during their first two measurement waves and self-reports during the last two. Different informants provide unique information that cannot be adjoined without first testing for measurement invariance across reporters. Finally, measurement invariance of the social withdrawal measure was not established prior to trajectory analysis. To date, no study has examined if the social withdrawal scales used were measurement invariant.

## Measurement Issues

We suspect that the limited progress of adolescent and adult withdrawal research is partly related to measurement obstacles such as possible informant biases and lack of measurement invariance. In the following sections, we will argue that self-report items from the Achenbach System of Empirically Based Assessment (ASEBA) provide an adequate method for obtaining social withdrawal ratings in adolescence and adulthood, and stress the importance of assessing measurement invariance in studies examining developmental patterns.

### Measuring Social Withdrawal in Adolescence and Adulthood

The majority of assessment measures and methods of social withdrawal focus on childhood, and only few have been developed or adapted for use in adolescent or adult samples. Furthermore, no questionnaire has been developed to measure the longitudinal changes in the global behavioral aspects of social withdrawal in adolescence and adulthood, such as shyness, spending excessive time alone, and avoiding peer interaction. To the best of our knowledge, only two validated proxy measures are available, the Behavioral Inhibition/Activation Scales (Coplan et al. 2006) and the Revised Cheek and Buss Shyness Scale (Cheek and Buss 1981), but these measures capture only inhibition toward novelty and shyness, respectively, rather than the global behavioral aspects that span across the types of social withdrawal. The Child Social Preferences Scale has been adapted for use with early adults to measure the three aspects of social withdrawal (i.e. shyness, unsociability, and avoidance; Nelson 2013). Although a promising new scale, more research is needed to determine its application in longitudinal research. A measure capturing the global behavioral aspects of social withdrawal, longitudinally, in adolescence and adulthood has been lacking.

Another issue that deserves attention concerns the validity of informant reports. Most instruments that assess social withdrawal in childhood obtain ratings from parents, teachers, or peers, but other-reports become less reliable in adolescence, when individuals spend more and more time outside parental supervision. Consequently, the difference between parent- and self-reports increases from childhood through adulthood (Van der Ende et al. 2012) due to decreasing parent-child contact. Similarly, obtaining teacher- or peer-ratings of withdrawal becomes more difficult when individuals take part in more flexible classes (i.e. attend secondary and tertiary education) or no longer belong to a formal education setting. A way to avoid these informant-related measurement problems is to assess social withdrawal by means of self-reports.

To overcome these two measurement issues, we need a self-report measure that captures the key characteristics of social withdrawal and is designed for longitudinal use during adolescence and adulthood. We suggest that the commonly used and well-validated ASEBA Youth Self-Report (YSR) and Adult Self-Report (ASR) could fulfil these criteria and overcome the limitations of previous studies. Both the YSR and the ASR contain a Withdrawn/Depressed scale that measures aspects of depression and social withdrawal. Several studies have already used this scale to assess social withdrawal. Among them, four have used the complete Withdrawn/Depressed scale (Katz et al. 2011; Lamb et al. 2010; Perez-Edgar et al. 2010; Rubin et al. 2013) and three used selected items from the scale, removing depression-related items to avoid confounding results (Booth-LaForce and Oxford 2008; Eggum et al. 2009; Tang et al. 2017). Importantly, only few of these studies spanned through the adolescent or early adult periods and none consistently used the YSR/ASR to assess the development of self-reported social withdrawal.

### Longitudinal Measurement Invariance

Items from the Withdrawn/Depressed scale have been reported to have high internal consistency, which is promising. However, it is unclear whether these items measure the same construct over time and show measurement invariance. Longitudinal measurement invariance (also called factorial invariance, measurement equivalence, or structural stability) of a variable is especially important when examining mean-level or individual trajectories. When examining longitudinal changes in social withdrawal, it is essential that the variable used captures the same aspects of withdrawal in the same way at every assessment wave. Although this may seem obvious at first glance, most studies do not examine measurement invariance prior to interpreting results, and few mention the possibility of measurement variance as a study limitation. Assessing longitudinal measurement invariance tells us if individuals interpret specific items of a given measure in the same way over time through a series of increasingly constrained Confirmatory Factor Analysis (CFA) models. Briefly, four types of measurement invariance, at increasing strength, are examined: configural invariance (baseline), metric invariance (weak), scalar invariance (strong), and residual invariance (strict), see methods section for details. Examining the longitudinal measurement invariance of social withdrawal is not only novel and timely but will provide information on the underlying structure of withdrawal across multiple developmental periods and, if social withdrawal is at least partially invariant, allow for valid interpretations of observed changes or trajectories in withdrawal over time.

## Overview of the Current Study

The current study uses 9 years of longitudinal data from a population-based cohort survey in order to fill some of the gaps in the literature and to answer four main questions: (1) Which withdrawal-related YSR and ASR items measure social withdrawal validly and reliably in our sample? (2) Is the structure of social withdrawal invariant over time? (3) What is the stability and normative change (i.e., mean-level continuity) of social withdrawal during adolescence and early adulthood? (4) How many trajectories of social withdrawal can be distinguished?

## Method

### Participants

Participants were part of the Tracking Adolescents’ Individual Lives Survey (TRAILS), a prospective, population-based cohort study aiming to track the social, psychological, and physical development of pre-adolescents through adulthood. During the first measurement wave in 2001 (T1), 2230 children (*M* age = 11.09, *SD* = 0.56; 50.8% female), who were born between October 1989 and September 1991, were recruited for participation. In subsequent waves, occurring every two or 3 years, 73–96% of the children from T1 participated again. More information about the recruitment and assessment procedure has been reported by De Winter et al. (2005), Huisman et al. (2008), and Oldehinkel et al. (2015). Extensive case analyses from T1 to T4 can be found in Nederhof et al. (2012). Participants who missed at least one measurement wave between T1 and T5 were more likely to be male, to come from low-socioeconomic families, and to have more externalizing problems at T1 (Oldehinkel et al. 2015). The current study uses data from the last four measurement waves (T3-T6). Due to missing social withdrawal data on every time point during T3 to T6, 313 participants were excluded from analyses, leading to a final sample size of 1917 adolescents (53% female; Table 1 depicts the retention rates and demographics).

**Table 1:** Participant demographics at each measurement wave

Wave	*M* age (SD) *N* = 1917	Survey N per wave	Surveyretention ^a^	Survey% female	Survey ethnicity %	
T3	16.26 (0.70)	1816	81%	52%	86.5%	Dutch
T4	19.06 (0.59)	1881	84%	52%	2.1%	Surinam
T5	22.28 (0.65)	1778	80%	53%	1.7%	Indonesian or Mollucan
T6	25.65 (0.60)	1617	73%	55%	1.7%	Antillean
					0.7%	Moroccan
					0.5%	Turkish
					6.9%	Other

Mean age (SD) are presented for the participants included in the current study (*N* = 1,1917) while the remaining columns present demographic data for the all participants in the larger survey (*N* = 2230)

^a^Survey retention refers to the proportion of participants from the baseline who participated in subsequent assessments

### Data Collection Procedure

The TRAILS study protocol was approved by the Dutch Central Committee on Research Involving Human Subjects. The adolescent participants of the study provided written consent at the second through sixth assessment waves. A parent or guardian provided written parental consent for adolescent participation during the first three assessment waves, and written consent to participate at every assessment wave.

At the initial assessment wave, well-trained interviewers visited one of the parents or guardians (95.6% mothers) at their home to conduct interviews regarding the family composition, child’s developmental history, somatic health, and impairments, health care use, and familial psychopathology. During this visit, parents also completed a written questionnaire. Children completed a questionnaire and neuropsychological tests at school, under the supervision of at least one TRAILS assistant. During the second and third assessment waves, parents completed a questionnaire, which they received via postal mail, and children completed a questionnaire at school, in groups, under TRAILS supervision. At the fourth assessment wave, a custom research company (CRC) was hired to recruit and assess participants, who were now over the age of 18 years, thereby requiring adolescent written informed consent but not parental consent for adolescent participation. Participants received information explaining that the CRC would collect data, and if participants gave informed consent to participate, the CRC sent them a web-based questionnaire battery. During the fourth wave, parents completed a questionnaire, which they received again via postal mail. During the fifth and sixth assessment waves, data collection was completed by the TRAILS team. During these waves, participants and parents received study information in print via mail, followed by an email or letter with the website link to the online questionnaire two to 3 weeks later. Reminders to complete the questionnaires were sent by email, followed by letters and/or telephone calls.

### Measures

#### Social Withdrawal

Social withdrawal was measured using items from the *Youth Self-Report* (YSR; Achenbach 2001) and *Adult Self-Report* (ASR; Achenbach 2003) Withdrawn/Depressed and Withdrawn scales, respectively. The YSR is a widely-used, 112-item self-report measure of emotional and behavioral problems, developed for adolescents aged 11 to 18 years. The items can be rated on a 3-point scale, with 0 = *not at all*; 1 = *a little or sometimes*; and 2 = *always or often true* in the past 6 months. The ASR is the adult version of the YSR, meant for individuals aged 18 to 59 years. The ASR includes 102 items rated on the same 3-point scale as the YSR. The YSR was administered at T1 to T3 and the ASR at T4 to T6. In a sample of 11- to 18-year-old youth, the YSR Withdrawn/Depressed scale had moderate 8-day test-retest reliability (*r* = 0.67), and scores were positively correlated with measures of depression (*rs >* 0.36, *p*s < 0.001) and withdrawal (*r*s > 0.58, *p*s < 0.001; Achenbach and Rescorla 2007). In a sample of adults over the age of 18 years, the ASR Withdrawn scale had high 7-day test-retest reliability (*r* = 0.87), and scores were positively correlated with measures of depression (*r* = 0.46, *p* < 0.01), anxiety (*r* = 0.44, *p* < 0.01), and social introversion (*r* = 0.43, *p* < 0.01; Achenbach and Rescorla 2003).

As a starting point for the analyses, we selected five withdrawal-related items, which were identical in the YSR and ASR: “*I would rather be alone than with others*,” “*I am secretive or keep things to myself*,” “*I am too shy or timid*,” “*I refuse to talk*,” and “*I keep from getting involved with others.*” Selection was based on face validity and on previous research (e.g., Booth-LaForce and Oxford 2008; Eggum et al. 2009; Katz et al. 2011; Tang et al. 2017). Cronbach’s alphas of the five items at T1 to T6 were 0.49, 0.57, 0.65, 0.67, 0.72, and 0.72, respectively. Although our original, preregistered plan included analyses of data from all six measurement waves, measurement invariance was not found when including T1 and T2, likely because of insufficient internal reliabilities (Cronbach’s alphas <0.60 given the few number of items; Loewenthal 2004) of the pre- and early adolescent responses. We therefore decided to perform all following analyses on T3 to T6 data, which showed sufficient reliabilities and measurement invariance over time.

#### Criterion Variables

***Shyness and social affiliation*** were assessed by the *Early Adolescent Temperament Questionnaire-Revised* (EATQ-R, Ellis and Rothbart 2001). The EATQ-R is a parent-report questionnaire measuring temperament in adolescents aged 9 to 15 years with 65 Likert-type items (1 = *Almost never true* to 5 = *Almost always true*). The scales of the EATQ-R include Fearfulness, Frustration, Shyness, Surgency, Affiliation, and Effortful Control, of which the Shyness and Affiliation scale were used for the purposes of this study. The Shyness scale measures hesitancy toward novel social situations, with items including “*My child is shy”* and “*My child is shy when he or she meets new people*.” The Affiliation scale measures the tendency to want closeness with others, including items such as “*My child likes talk to someone about everything he or she thinks”* and “*My child would like to spend time with a good friend every day.”* In a sample of early adolescents, the Shyness scale had good 8-week test-retest stability (intra-class correlation = 0.73), and scores were positively correlated with two measures of behavioral inhibition (*r*s = 0.39 and 0.45, *p*s < 0.001) and with measures of anxiety, depression, and emotional problems (*r*s = 0.34, 0.25, and 0.34, *p*s < 0.001, respectively; Muris and Meesters 2009). In the same sample, the Affiliation scale had good test-retest stability (intra-class correlation = 0.80), and scores were correlated with a measure of prosocial behavior (*r* = 0.39, *p* < 0.001). The EATQ-R was completed by parents at T3, T4, and T5 with acceptable internal consistencies for both Shyness (4 items, α = 0.87, 0.78, 0.80) and Affiliation (5 items, α = 0.73, 0.63, 0.66).

***Reduced social contact*** was measured by the 12-item Reduced Social Contact scale of the *Children’s Social Behavior Questionnaire* (CSBQ) and *Social Behavior Questionnaire-Adult Version* (SBQ-A; Hartman et al. 2006). The CSBQ and SBQ-A were developed to assess the socio-behavioral symptoms of Autism Spectrum Disorders. The CSBQ is a parent-report measure consisting of 49 items rated on a three-point scale (0 = *Never*; 1 = *A little/sometimes*; 2 = *Often*). The SBQ-A parent-report form is the adult version of the CSBQ, with 44 items rated on the same three-point scale. The CSBQ Reduced Social Contact scale was administered at T3 and T4 (α = 0.89, 0.86); at T6, the SBQ-A Reduced Social Contact Scale was administered (α = 0.77). The Reduced Social Contact scale includes 12 items such as “*Does not start playing with other children*”,” *Has little or no need for contact with others*”, and “*Does not respond to other children’s initiatives*”.

***Antisocial behaviors*** were assessed with the *Antisocial Behavior Questionnaire* (ASBQ; Moffitt and Silva 1988). The ASBQ is a self-report questionnaire assessing the frequency of antisocial behaviors (e.g., theft, truancy, substance use) in the past 12 months. Items are rated on a 5-point scale (0 = *no/never*, 1 = *one time*, 2 = *two to three times*, 3 = *four to six times*, 4 = *seven or more times*). The ASBQ consisted of 25 items at T3 (α = 0.86), and 26 items at T4, T5, and T6 (α = 0.79, 0.74, 0.69).

***Anxiety*** was assessed by the YSR (Achenbach 2001) and ASR (Achenbach 2003) Anxious/Depressed subscale. The YSR Anxious/Depressed subscale included 13 items at T3 (α = 0.84) and the ASR Anxious/Depressed subscale included 18 comparable items at T4-T6 (α = 0.91, 0.92, 0.93). Because of the discrepancy between the number of items, our analyses utilized the mean Anxiety per participant, based on item endorsement, rather than the total Anxiety score.

## Statistical Analyses

Analyses were conducted in MPlus Version 8.0 (Muthén & Muthén 1998-2017) using maximum likelihood with robust standard errors (MLR) estimation. First, a CFA model with five YSR items loading onto a single social withdrawal latent variable during baseline (T3) was tested in half of the data. The following goodness of fit cutoffs were considered to indicate a good model fit: comparative fit index (CFI) ≥ 0.95, root mean square error of approximation (RMSEA) ≤ 0.06, and standardized root mean square residual (SRMR) ≤ 0.08 (Hu and Bentler 1999). If the model had good fit, analyses were repeated in the second half of the data.

Next, a series of increasingly constrained CFA models systematically tested if the YSR/ASR items, indicating a single social withdrawal latent factor, were measurement invariant over time. Differences in model fit were examined in two ways: first, using the Satorra-Bentler scaled chi-square difference tests (ΔSBχ^2^; Satorra and Bentler 2001), and second, using change-in-fit indices following the Chen (2007) criteria: ΔCFI ≥ −0.010, ΔRMSEA ≥0.015, and ΔSRMR ≥0.030 for metric invariance, and ΔSRMR ≥0.010 for scalar invariance, because SRMR is less sensitive to noninvariance in intercepts than noninvariance in item loadings. Priority was given to the chi-square difference test for determining if the data demonstrated invariance (Bowen and Masa 2015; Vandenberg and Lance 2000), with further support for model fit conclusions based on the change-in-fit indices (Chen 2007). In the configural invariance model, factor loadings, intercepts, and residual variances were allowed to vary. Factor loadings were constrained to be equal over time in the metric model, and factor loadings and item intercepts were constrained to be equal in the scalar model. If the scalar model fits significantly worse than the metric model, up to 20% of intercepts were allowed to vary until the model fitted the data as well as the metric model. When this happens, partial scalar invariance is established. Within the (partial) scalar invariance model, the social withdrawal latent variable correlations between consecutive time points provide information about the rank-order stability of social withdrawal.

After that, we examined the normative mean-level change in social withdrawal over time. A multiple-indicator Latent Growth Model (mLGM) assessed the growth curve of the social withdrawal latent variable. This model was preferred over traditional item summation scores, because mLGMs account for both random and systematic variance through the use of latent variables. Furthermore, the mLGM included results from the measurement invariance analyses, and hence prevented biasing growth results with any metric discrepancies. The mLGM included (1) the measurement model, which defined social withdrawal from the five YSR/ASR items and specified factor loadings and intercept equalities found in the measurement invariance analyses, and (2) the intra-individual linear or quadratic changes in social withdrawal over time, defined by the intercept growth factor, linear slope growth factor, and the quadratic slope factor latent variables. The variance of the intercept and slope growth factors indicated the amount of individual differences at baseline and in trajectories, respectively.

Finally, we extended the mLGM to determine the number and type of distinct linear and quadratic social withdrawal trajectory classes by assessing how the data fitted one- to four-class models through multiple-indicator Latent Class Growth Analysis (mLCGA). To determine the best class enumeration, we used the Lo-Mendell-Rubin adjusted Likelihood Ratio Test (aLRT) and the Bayesian Information Criterion (BIC). The aLRT compares *k*-1 class model to the *k*-class model; a significant value indicates that the *k* class fits the data better than the *k*-1 class model. Additional evidence for the number of latent classes was provided by Akaike’s Information Criterion (AIC), the sample size adjusted BIC (SSBIC), and the entropy of the model.

## Results

### Item Selection for Social Withdrawal Latent Variable

The CFA model of five items loading on a single latent withdrawal factor on half of the T3 data demonstrated excellent fit: *X*^2^(5) = 21.420 *p* < 0.001; RMSEA = 0.062, 90% CI [0.037, 0.091]; SRMR = 0.028; CFI = 0.961. Results were replicated in the second half of the data: *X*^2^(5) = 23.381 *p* < 0.001; RMSEA = 0.063, 90% CI [0.037, 0.092]; SRMR = 0.029; CFI = 0.956. All five YSR/ASR items were thus retained for the remaining analyses.

### Longitudinal Measurement Invariance

Results from the longitudinal measurement invariance models are depicted in Table 2. First, we specified a configural invariance model in which all factor loadings, intercepts, and residual variances are allowed to vary. Factor variances were all fixed to 1 and all factor means were fixed to 0 for model identification. Results indicated that the configural invariance model was an excellent fit to the data. Next, we specified a metric invariance model in which the factor loadings were constrained to be equal over time, while intercepts and residual variances were allowed to vary. The withdrawal factor variance at T3 was fixed to 1 and all factor means were fixed to 0 for identification. Results indicated that the metric invariance model had an excellent fit to the data too, and was no worse than the configural invariance model. Finally, we specified a scalar invariance model, which constrains all factor loadings and intercepts to be equal while allowing residual variances to vary. The withdrawal factor variance at T3 was fixed at 0 and the factor mean at time 1 was fixed at 0 to allow model identification. The scalar invariance model fit the data significantly worse compared to the metric model. Using modification indices to determine which factor loadings needed to be freed to improve model fit, we freed intercepts one-by-one in testing partial scalar invariance. Comparisons between the partial scalar model and metric model were made until the scalar invariance model did not fit the data significantly worse than the metric model. We freed four item intercepts (20% of the intercepts) to achieve partial scalar invariance (“*I am too shy or timid*” and “*I keep from getting involved with others*” at T3; “*I’d rather be alone than with others*” at both T5 and T6). Although the SB-scaled *X*^2^ difference test was still significant (*p* = 0.02), the strictest change in fit-index criteria were met, indicating that the metric and partial scalar model with four freed intercepts fit the data almost identically well.

**Table 2:** Results from the assessment of longitudinal measurement invariance

	X^2^ (df)	SBΔX^2^ (Δdf)	SB ΔX^2^*p* value	CFI	ΔCFI	RMSEA [90% CI]	ΔRMSEA	SRMR	ΔSRMR
Configural	219.244 (134)			0.986		0.018 [0.014, 0.022]		0.025	
Metric	234.182 (146)	15.464 (12)	0.217	0.986	0.000	0.018 [0.013, 0.022]	0.000	0.027	0.002
Scalar	548.207 (158)	382.676 (12)	0.000	0.937	−0.049	0.036 [0.033, 0.039]	0.018	0.038	0.011
Scalar partial 1^a^	431.045 (157)	237.621 (11)	0.000	0.955	−0.031	0.030 [0.027, 0.034]	0.012	0.035	0.008
Scalar partial 2^a^	352.631 (156)	107.449 (10)	0.000	0.972	−0.014	0.024 [0.020, 0.027]	0.006	0.030	0.003
Scalar partial 3^a^	267.571 (155)	37.734 (9)	0.000	0.982	−0.004	0.019 [0.015, 0.023]	0.001	0.028	0.001
Scalar partial 4^a^	251.131 (154)	17.908 (8)	0.022	0.984	−0.002	0.018 [0.014, 0.022]	0.000	0.027	0.000

*SB*, Satorra-Bentler scaled; *CFI*, comparative fit index; *RMSEA*, root mean square error of approximation; *SRMR*, standardized root mean residual

^a^Model comparisons are made with the metric invariance model

### Rank-Order Stability of Social Withdrawal

Rank-order stability of social withdrawal was determined by the correlations between consecutive withdrawal latent factors, and indicated substantial stability over time: *r*_T3-T4_ = 0.70; *r*_T4-T5_ = 0.67; *r*_T5-T6_ = 0.72; all *p*s < 0.001.

### Mean-Level Change of Social Withdrawal across the Full Sample

**Fig. 1:**
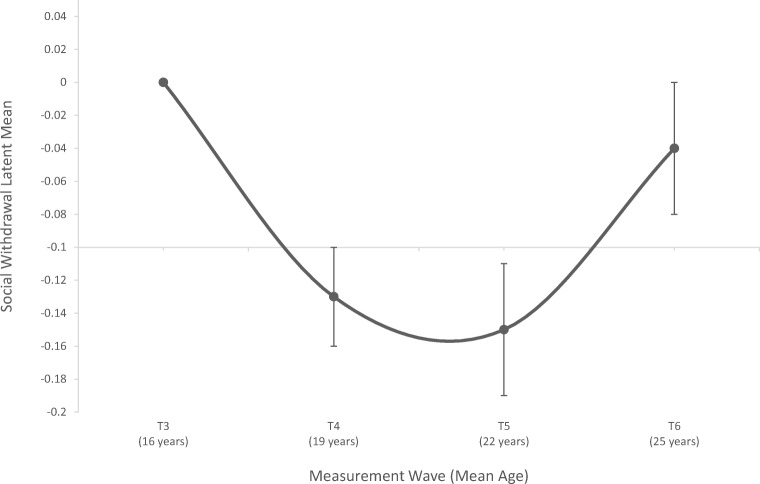
The mean-level longitudinal trajectory of social withdrawal. Points represent the estimated latent means from the multiple-indicator latent growth curve analysis

We could examine both linear and quadratic changes in social withdrawal because we had four time points of data. In our mLCG model, factor loadings were constrained to be equal across time (except for the first item in every time point, constrained to 1, for model identification), item intercepts were constrained to be equal across time except the intercepts which were freed in the partial scalar model, and residual errors were correlated. The means and standard errors (SE) of the social withdrawal latent variables were: *M*_T3_ = 0 (0); *M*_T4_ = −0.13 (0.03); *M*_T5_ = −0.15 (0.04); *M*_T6_ = −0.04 (0.04). The quadratic model had an excellent fit, *X*^2^(155) = 251.529, *p* < 0.001; RMSEA = 0.018, 90% CI [0.014, 0.022]; SRMR = 0.027; CFI = 0.984. The mean quadratic slope growth factor was positive and significant (estimate = 0.019, *p* < 0.001; Fig. 1). Social withdrawal steeply decreased from T3 to T5 and increased again from T5 to T6. The intercept variance was significant (intercept variance = 0.068, *p* < 0.001) while the quadratic slope variance was non-significant (quadratic slope variance = 0.002, *p* = 0.277), indicating that there was a significant U-shaped trajectory in the overall sample with individual differences in baseline levels of social withdrawal.

### Distinct Social Withdrawal Trajectories

**Fig. 2:**
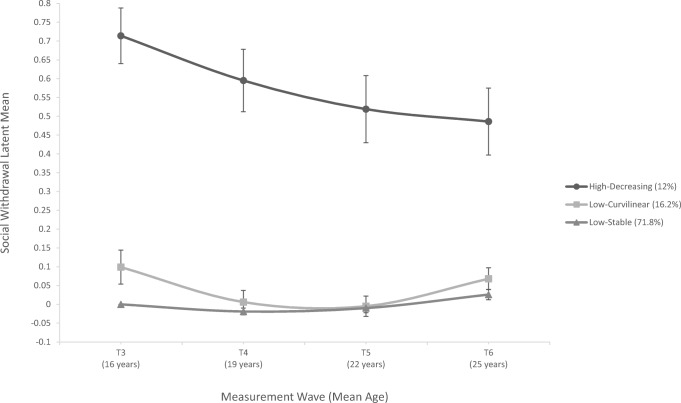
Longitudinal trajectories of social withdrawal. Points represent the estimated latent means from the multiple indicator latent class growth analysis for a three-class solution

Multiple-indicator LCGA identified three trajectories of social withdrawal: a low-stable group (71.8%), a high-decreasing group (12.0%), and a low-curvilinear group (16.2%; Fig. 2). Table 3 depicts the multiple-indicator LCGA results and Table 4 depicts the parameter estimates of the intercept and slope factors, and their respective variance estimates, of the three classes. The majority of participants were classified into the low-stable group with the lowest levels of withdrawal throughout the four measurement waves. The high-decreasing withdrawal group had the highest level of social withdrawal at every time point, but demonstrated a linear decrease over time. Finally, the low-curvilinear group had baseline levels of withdrawal between the low-stable and decreasing groups, decreased to the low-stable levels of withdrawal during the second and third measurement waves, and had a slight increase in withdrawal during the final wave.

**Table 3:** Criteria of multiple-indicator latent class growth analyses for one- to four-class solutions

Latent classes	Loglikelihood	Parameters	AIC	BIC	SSABIC	Entropy	aLMR k-1 vs. k classes
1	−18,851.053	75	37,852.106	38,268.994	38,030.719		
2	−17,714.372	82	35,592.744	36,048.543	35,788.028	0.902	*p* = 0.26 (1 > 2)
3	−17,503.913	90	35,187.825	35,688.092	35,402.161	0.884	*p* = 0.03 (2 < 3)
4	−17,525.317	98	35,246.635	35,791.369	35,480.022	0.868	*p* = 0.65 (3 > 4)

*AIC*, Akaike’s Information Criterion; *BIC*, Bayesian Information Criterion; *SSABIC*, sample size adjusted Bayesian Information Criterion; aLMR, Lo-Mendell-Rubin Adjusted Likelihood Ratio Test

**Table 4:** Parameter estimates of the intercept and slope factors, and their respective variance estimates, of the three trajectory classes

	High-decreasing (*n* = 221)			Low-Curvilinear (*n* = 276)			Low-Stable (*n* = 1420)		
Parameters	*M*	SE	σ^2^	*M*	SE	σ^2^	*M*	SE	σ^2^
Intercept	0.714***	0.074	0.010***	0.099*	0.045	0.010***	0.000	–	0.010***
Linear Slope	−0.141**_a_	0.066	0.029***	−0.135** _a_	0.045	0.029***	−0.033**	0.012	0.029***
Quadratic Slope	0.022	0.020	0.002	0.041***	0.013	0.002	0.014***	0.004	0.002

Means with same subscript do not significantly differ from one another. Means without a subscript are significantly different from one another at *p* < 0.05. The mean and standard error (SE) of the intercept parameter of the intercept factor of the low-stable trajectory was set to zero for model identification

*p < 0.05 **p < 0.01 ***p < 0.001

### Post-Hoc Analyses: Differences between Trajectory Groups on Associated Variables

Once we identified the three trajectories of social withdrawal, we were interested in exploring how these groups differed. We selected six relevant variables: gender, antisocial behavior, anxiety, shyness, affiliation, and reduced social contact. Gender, antisocial behavior, and anxiety were selected because the relationships between these variables and social withdrawal are commonly examined in the preadolescent literature, but little is known about these associations during adolescence and adulthood. Shyness, affiliation, and reduced social contact were selected as measures pointing to individuals’ more specific withdrawal characteristics. Shyness captures a hesitancy toward novel social situations; affiliation is the extent to which close relationships are desired; and reduced social contact measures the underlying social disinterest toward peers.

A chi-square test indicated that gender was not equally distributed among the three groups, χ^2^ (2, *N* = 1917) = 18.33, *p* < 0.001. The low-curvilinear group had a significantly higher proportion of males (58.7%) compared to the low-stable (44.6%) and high-decreasing (47.5%) groups. To examine class differences on the withdrawal-related variables, while controlling for gender, accounting for classification error, and keeping the class distributions the same as in the three-class LCGA model, we used the three-step BCH approach (Asparouhov and Muthén 2014; Bolck et al. 2004; Vermunt 2010). This method accounts for classification error of class membership by using posterior probabilities to weigh the likelihood of each trajectory class membership. The manual BCH approach in MPlus (Asparouhov and Muthén 2014; Muthén & Muthén 1998-2017) allowed us to include both covariates (i.e. gender) and distal outcomes (i.e. withdrawal-related variables) that are predicted by class membership. The BCH analysis provided pairwise differences in the weighted means of shyness, affiliation, reduced social contact, antisocial behaviors, and anxiety, while controlling for gender, using a Wald chi-square test per variable per time point. Table 5 depicts the weighted means and standard deviations of the withdrawal-related variables, stratified by classes of withdrawal trajectory, after accounting for imprecision of class membership, and the results from the Wald chi-square tests and pairwise comparisons.

**Table 5:** Means and standard deviations of withdrawal-related variables across measurement waves, after accounting or imprecision of class membership and controlling for gender, stratified by classes of withdrawal trajectory

	Low-Stable (*n* = 1420) *M* (*SD*)	Low-Curvilinear (*n* = 276) *M* (*SD*)	High-Decreasing (*n* = 221) *M* (*SD*)	Wald χ^2^	Df	*p* Value	Pairwise comparisons
Shyness							
T3	2.30 (0.90)	2.32 (0.87)	2.77 (0.97)	23.045	2	<0.001	HD > LS, C
T4	2.00 (0.81)	2.01 (0.74)	2.42 (0.96)	24.669	2	<0.001	HD > LS, C
T5	1.84 (0.75)	1.90 (0.73)	2.35 (0.91)	37.996	2	<0.001	HD > C > LS
Affiliation							
T3	3.76 (0.61)	3.62 (0.63)	3.31 (0.66)	16.657	2	<0.001	LS, C > HD
T4	3.60 (0.57)	3.49 (0.61)	3.33 (0.65)	10.592	2	0.005	LS > C > HD
T5	3.75 (0.55)	3.62 (0.55)	3.53 (0.65)	4.386	2	0.11	–
Antisocial Behavior							
T3	0.20 (0.28)	0.29 (0.32)	0.25 (0.33)	10.172	2	0.006	C > LS
T4	0.07 (0.15)	0.08 (0.13)	0.13 (0.25)	3.442	2	0.18	–
T5	0.05 (0.11)	0.07 (0.11)	0.09 (0.18)	6.917	2	0.03	HD > LS
T6	0.04 (0.10)	0.05 (0.10)	0.06 (0.12)	5.024	2	0.08	–
Reduced Social Contact							
T3	0.15 (0.23)	0.18 (0.26)	0.36 (0.33)	25.994	2	<0.001	HD > LS, C
T4	0.15 (0.24)	0.17 (0.21)	0.37 (0.41)	27.836	2	<0.001	HD > LS, C
T6	0.10 (0.21)	0.07 (0.15)	0.32 (0.37)	23.191	2	<0.001	HD > LS, C
Anxiety							
T3	3.20 (3.19)	3.81 (3.59)	7.42 (4.99)	133.375	2	<0.001	HD > C > LS
T4	5.03 (5.54)	4.39 (4.18)	11.81 (6.40)	97.195	2	<0.001	HD > LS > C
T5	4.81 (5.41)	4.75 (5.31)	11.70 (7.74)	83.850	2	<0.001	HD > LS > C
T6	6.43 (6.48)	6.07 (5.44)	12.33 (7.96)	51.613	2	<0.001	HD > LS, C

Mean age of participants was 16.3 years at T3, 19.1 years at T4, 22.3 years at T5, and 25.7 at T6

Results indicated significant class differences on shyness, reduced social contact, and anxiety at every time point, on affiliation during two out of three time points, and on antisocial behaviors during two out of four time points. Pairwise comparisons indicated that the high-decreasing class was significantly shyer than the low-stable and low-curvilinear classes at every time point. At T3 and T4, the low-stable and low-curvilinear class did not differ on shyness, but at T5 the low-curvilinear group was significantly shyer than the low-stable class. At T3 and T4, the high-decreasing class had the lowest affiliation. At T3, the low-stable and low-curvilinear class did not differ in affiliation, but at T4, the low-curvilinear class reported significantly lower affiliation compared to the low-stable class. The three classes no longer differed on affiliation at T5. Classes also differed on antisocial behaviors at T3 and T5, but did not differ at T4 and T6. During T3, the low-curvilinear group endorsed more antisocial behaviors than the low-stable class, while the high-decreasing class did not differ from the other two. At T5, the high-decreasing class endorsed more antisocial behaviors than the low-stable class, while the low-stable and low-curvilinear classes did not differ. The high-decreasing class had the highest reduced social contact at every time point, while the low-curvilinear and low-stable groups did not differ. Finally, the high-decreasing class also reported the highest anxiety at every time point. The low-curvilinear class reported higher anxiety than the low-stable class at T3; the low-stable class reported higher anxiety than the low-curvilinear class at T4 and T5; and at T6, the low-stable and low-curvilinear groups did not differ on anxiety.

## Discussion

The study presented in this article used almost a decade of longitudinal data to examine the mean-level change and specific trajectories of social withdrawal through adolescence and early adulthood. We contributed to the small, but expanding, adolescent and early adult social withdrawal literature in hopes of increasing our knowledge of the normative and at-risk patterns of withdrawal during this transitional period of life. Prior to examining the trajectories of social withdrawal, we aimed to overcome some of the measurement-related limitations in previous studies, such as informant biases and possible measurement noninvariance, by examining the ability for self-report measures to capture withdrawal in the same way over time. We found evidence that the YSR and ASR can be used through adolescence and early adulthood to assess global behavioral aspects of social withdrawal, such as shyness, spending excessive time alone, and avoiding peer interaction. The five selected withdrawal-related items captured a single dimension of social withdrawal and were partially measurement invariant over time. This indicates that the selected withdrawal items were interpreted in the same way between the ages of 16 and 25. We could not establish measurement invariance when including data from measurement waves prior to the age of 16 years, indicating that the interpretations of the withdrawal items were different in pre- and early adolescence compared to middle and late adolescence and young adulthood. This is important because previous studies have made conclusions about the trajectories of social withdrawal with broad age ranges spanning from childhood through adulthood without examining the measurement invariance of their withdrawal items. The validity of these conclusions is questionable considering the changing interpretations of items during adolescence. With confidence, we can interpret our subsequent findings in participants aged 16 to 25 as real developmental changes rather than as measurement artifacts.

### Normative Mean-Level Withdrawal Changes

Results did not support our hypothesis that the mean-level change of social withdrawal follows a curvilinear, inverted-U trajectory. On the contrary, we found a U-shaped curvilinear trajectory, in which social withdrawal decreased from 16 to 19 years (T3-T4), remained low and stable from 19 to 22 years (T4-T5), and increased again from 22 to 25 years (T5-T6). This curvilinear pattern of social withdrawal might be related to the changes in individuals’ social relationships during late adolescence and again during early adulthood. The decrease in mean-level social withdrawal from 16 to 19 years might be driven by the increasing size of individuals’ social network during the same time. The size of the social network increases during late adolescence (Wrzus et al. 2012), due to increasing social motivations, greater autonomy from parents, and the entry to postsecondary institutions which expose individuals to a large number of new peers. These changes mean more opportunities for socializing, forming new relationships, and expanding one’s social network. The increasing social network size likely underlies the decrease in social withdrawal from 16 to 19 years and the maintenance of low levels of withdrawal from 19 to 22 years.

Similarly, the increase in mean-level social withdrawal from 22 to 25 years might be driven by decreasing sizes of individuals’ social networks. The size of the social network of adolescents increases until early adulthood, then begins to steadily decrease (Wrzus et al. 2012). This social network decline is due to common life events during early adulthood such as exiting post-secondary education, entering the job market, transitioning to parenthood, and/or relocating. These life events lead to fewer people in the social network of early adults, thereby limiting opportunities for social experiences and contributing to early adults’ perceptions of themselves as more withdrawn.

In sum, the U-shaped mean-level trajectory of social withdrawal during adolescence and early adulthood is probably related to the changes in the social network during these ages. Future studies should examine the longitudinal relationship between social network changes and the social withdrawal trajectory during adolescence and early adulthood. Furthermore, more frequent assessments of the size and changes of the social network over a short period of time during the observed withdrawal decreases (16 to 19 years) and increases (22 to 25 years) could offer insights into the underlying mechanisms of the network-withdrawal relationship.

### Three Trajectories of Social Withdrawal

We found three distinct withdrawal trajectory groups: a low-stable group (71.8%), a high-withdrawal group (12%), and a low-curvilinear group (16.2%). Most individuals had consistently low levels of withdrawal, which was expected considering that most community cohort studies report low levels of withdrawal or other problem behavior. Our post-hoc analyses indicated that this group was well adjusted, with high initial levels of social affiliation and low initial levels of shyness, antisocial behaviors, reduced social contact, and anxiety. Furthermore, we found that even in this majority low-stable group social withdrawal increased from 22 to 25 years, providing further support for a normative increase in withdrawal during early adulthood.

The low-curvilinear group had higher withdrawal than the low-stable group when they were aged 16 and 25 years, but was no different from the low-stable group from 19 to 22 years. Notably, the social withdrawal levels of the low-curvilinear group deviated substantially enough from the low-stable group to distinguish these individuals as following a distinct trajectory. The higher withdrawal of the low-curvilinear group at 16 years could be due to unsociable or avoidant tendencies of these adolescents.). At age 16, the low-curvilinear group endorsed more frequent participation in antisocial behaviors than the low-stable group, indicating higher externalizing behaviors which may have contributed to withdrawal via peer exclusion. These results may indicate that externalizing youth become less withdrawn during late adolescence and early adulthood due to decreases in externalizing behaviors, which promote greater peer acceptance (Bongers et al. 2003). The low-curvilinear group was also more withdrawn than the low-stable group at 25 years. This increase likely reflects the normative increase in withdrawal in early adulthood that was discussed previously, but the reason why the low-curvilinear group surpassed the withdrawal levels of the low-stable group after being at identical withdrawal levels for years is unknown. Future research should focus on non-anxious withdrawn adolescents, such as those with unsociable, avoidant, or externalizing characteristics. Our results point to the possibility that these individuals decrease in withdrawal during late adolescence and early adulthood, but further investigation to the reasons behind this decrease (e.g. greater sensitivity to social network changes) is warranted.

Finally, a considerable percentage of individuals were persistently withdrawn through adolescence and early adulthood. The high-decreasing group reported the highest shyness, reduced social contact, and anxiety, and the lowest affiliation, at every time point, indicating that the high-decreasing group was the most maladjusted. Although this high-decreasing group had decreasing levels of withdrawal over time, they were considerably more withdrawn compared to those in the other two groups at every time point. The decrease in withdrawal could be due to the establishment of new relationships, albeit at a slower rate, or age-related improvements in social or coping skills. Regardless, withdrawal in this group might be maintained by a negative feedback loop described by Rubin et al. (2009). Withdrawn youth avoid interacting with peers, which limits opportunities to develop social skills. Limited social skills contribute to withdrawn behavior during peer interactions, which elicit negative feedback from peers. Negative peer feedback perpetuates negative self-beliefs and anxiety, leading to greater withdrawal. Through this cyclical process, socially withdrawn adolescents are unable to follow the normative social network expansion during adolescence. Relatedly, withdrawn individuals are at greater risk for psychopathology (e.g. anxiety, depression), which could further maintain withdrawal during adolescence and early adulthood. The specific factors and mechanisms that maintain these high levels of social withdrawal during adolescence and early adulthood remain unknown. Future studies should examine the mechanisms that maintain high levels of social withdrawal in some adolescents. One possible mechanism is anxiety, which is theorized to underlie the negative feedback loop mentioned previously. The relationships between social withdrawal and anxiety are still poorly understood (Kingerly et al. 2010) and more research is needed to determine how withdrawal and anxiety influence one another in perpetuating high levels of one another (Perez-Edgar and Guyer 2014).

Three withdrawal trajectories have been reported in previous studies. Apart from the current study, the only other study to examine the trajectories of social withdrawal in adolescents and early adults was by Tang and colleagues (Tang et al. 2017). They found three trajectories in participants ages 8 to 35 years, two trajectories of which were different from the trajectories found in our study. Consistent with Tang et al., as well as with the preadolescent literature (Booth-LaForce et al. 2012; Booth-LaForce and Oxford 2008; Eggum et al. 2009; Oh et al. 2008), we found that most individuals had low-stable levels of withdrawal over time. Inconsistently, we found a high-decreasing and a low-curvilinear trajectory instead of linear increasing and decreasing groups. These inconsistencies are related to the differences in how we conceptualized and measured social withdrawal. First, we used only self-reports to capture social withdrawal at every assessment wave while Tang and colleagues used self- and parent-reports at different ages. Different levels of withdrawal symptoms are found when using ratings from different informants (Rubin et al. 2013) due to the context in which behaviors are observed. Parents may underestimate the social interaction and social involvement of their children as youth become increasingly autonomous during adolescence or no longer live in the parental home in early adulthood. This underestimation could inflate withdrawal estimates, thereby influencing the shapes of the trajectories. Second, we included participants who were in a specific period of development, namely adolescence and early adulthood. Perhaps by focusing on these two short and adjacent developmental periods, we captured withdrawal processes which were more time-limited compared to the broader developmental periods included in Tang et al. Notably, Tang et al. included withdrawal assessments during 12–16 years and 22–26 years, leaving a gap during the major transition from adolescence to early adulthood (i.e. 16 to 22 years). Our study filled this developmental gap and zoomed in on the withdrawal patterns during this transition, thereby creating differences in the trajectory shapes from previous studies.

### Strengths and Limitations

The current study was the second to include assessment points during adolescence and early adulthood when examining the longitudinal trajectories of social withdrawal, and the first to include multiple assessment waves during this transitional period to capture more fine-grained withdrawal changes during these ages. Prior to trajectory analyses, we established partial measurement invariance of the YSR and ASR withdrawal items. No previous study examined the measurement properties of these items in relation to developmental changes. In doing so, we have obtained further psychometric support for the use of the YSR and ASR withdrawal items, allowing for more social withdrawal research using these measures. Furthermore, our longitudinal design allowed us to examine curvilinear patterns of withdrawal, both at the mean and individual levels. Through our longitudinal design, we could also examine how trajectory groups differed on the same variable (i.e., shyness, affiliation, antisocial behaviors, etc.) over time, providing preliminary insights into the magnitude and stability of these group differences. Overall, this study contributed to the expanding literature on adolescent and early adult social withdrawal and increased our understanding of the normative and at-risk expression of withdrawn behaviors during these ages.

Results should be interpreted with consideration of several limitations. First, we used a global conceptualization of social withdrawal, which did not distinguish individuals based on underlying motivations to withdrawal. The five YSR and ASR items captured global behavioral characteristics of withdrawal such as shyness, preference for solitude, and refusal to talk; although they loaded on a single withdrawal latent factor, the underlying reason for endorsing an item can vary widely. An individual may “*keep from getting involved with others*” because they fear negative evaluation, because they are disinterested in others, or because they are excluded or neglected by their peers. Different underlying motivations to withdrawal contribute to different types of maladjustment (Rubin et al. 2009). Future studies are advised to examine the motivation to withdrawal and if underlying motivations change over time. A second limitation is that there was some overlap between the ages of our participants in adjacent assessment waves. This may have increased the standard errors because older individuals could differ from younger individuals within an assessment wave, but it is unlikely that this within-wave heterogeneity has caused systematic bias. Future studies could model withdrawal trajectories more sensitively with more homogenous age groups or more frequent assessment waves. Third, we did not include T1 and T2 data because measurement invariance was not found when including these time points, possibly due to the low internal reliability of social withdrawal items during these time points. On one hand, this means that subsequent results were robust and reflected real developmental changes; on the other hand, perhaps we have applied stricter invariance criteria than necessary to draw valid conclusions about the invariance and exclusion of younger ages. The reasons behind and the developmental implications of non-invariance of the withdrawal scores at younger ages are beyond the scope of this study, but seems worthy of exploration in future studies. Fourth, we relied solely on self-reported social withdrawal. Although we established measurement invariance of the self-reported social withdrawal items and believe self-reports of withdrawal are more suitable for early adulthood than other-reports, a multi-informant approach might capture withdrawal more validly and across multiple settings. Future studies should aim to include additional informants, such as parents, romantic partners, or observations, when examining social withdrawal in early adulthood. Finally, the large majority of participants were from an ethnically Dutch background, and participants from minority groups were heterogeneous. This prevented us from examining ethnic differences in social withdrawal trajectories. Future studies might include a more ethnically diverse sample to examine if minority group status is a risk or protective factor for social withdrawal during early adulthood.

## Conclusion

This study investigated the mean-level and individual trajectories of social withdrawal during adolescence and early adulthood. We found that the normative pattern of social withdrawal during these ages follows a U-shaped curve, with the lowest levels during late adolescence, and that individuals follow three withdrawal trajectories. Although most maintained low levels of social withdrawal throughout adolescence and early adulthood, 12% of individuals were persistently withdrawn. These results indicate that social withdrawal continues to be a developmentally relevant behavior after childhood, impacting the lives of adolescents and young adults. Many questions remain about the roles and mechanisms of social withdrawal during adolescence and adulthood.

## Electronic supplementary material

Table S1 (DOCX 17 kb)
