# Supplementary material for: Social Withdrawal in Adolescence and Early Adulthood: Measurement Issues, Normative Development, and Distinct Trajectories
Source: J Abnorm Child Psychol. 2018 Nov 27;47(5):865–79. doi: 10.1007/s10802-018-0497-4 (PMC6469640; doi:10.1007/s10802-018-0497-4)
Supplement: Supplementary file 1 — (DOCX 17 kb) [file 10802_2018_497_MOESM1_ESM.docx]

**Supplementary Materials**

Table S1.

*Configural, metric, and partial scalar invariance model factor loadings, intercepts, and residual variances*

| YSR/ASR item number and content | Loadings | | | |  | Intercepts | | | |  | Residual variances | | | |
| --- | --- | --- | --- | --- | --- | --- | --- | --- | --- | --- | --- | --- | --- | --- |
|  | T3 | T4 | T5 | T6 |  | T3 | T4 | T5 | T6 |  | T3 | T4 | T5 | T6 |
| **Configural Invariance Model** |  |  |  |  |  |  |  |  |  |  |  |  |  |  |
| 42/36: I would rather be alone than with others | 0.311 | 0.304 | 0.345 | 0.374 |  | 0.421 | 0.378 | 0.435 | 0.587 |  | 0.204 | 0.195 | 0.191 | 0.226 |
| 65/55: Refuse to talk | 0.178 | 0.168 | 0.169 | 0.175 |  | 0.141 | 0.115 | 0.113 | 0.136 |  | 0.105 | 0.092 | 0.083 | 0.103 |
| 69/59: I am secretive or keep things to myself | 0.424 | 0.454 | 0.480 | 0.468 |  | 0.620 | 0.577 | 0.548 | 0.596 |  | 0.262 | 0.277 | 0.238 | 0.232 |
| 75/64: I am too shy or timid | 0.286 | 0.283 | 0.280 | 0.293 |  | 0.572 | 0.366 | 0.337 | 0.358 |  | 0.315 | 0.248 | 0.218 | 0.235 |
| 111/91: I keep from getting involved with others | 0.249 | 0.216 | 0.249 | 0.283 |  | 0.295 | 0.147 | 0.169 | 0.203 |  | 0.176 | 0.104 | 0.111 | 0.120 |
| **Metric Invariance Model** |  |  |  |  |  |  |  |  |  |  |  |  |  |  |
| 42/36 I would rather be alone than with others | 0.319 | 0.319 | 0.319 | 0.319 |  | 0.421 | 0.378 | 0.435 | 0.587 |  | 0.202 | 0.193 | 0.193 | 0.233 |
| 65/55 I refuse to talk | 0.166 | 0.166 | 0.166 | 0.166 |  | 0.141 | 0.115 | 0.113 | 0.136 |  | 0.107 | 0.092 | 0.083 | 0.102 |
| 69/59 I am secretive or keep things to myself | 0.439 | 0.439 | 0.439 | 0.439 |  | 0.620 | 0.577 | 0.548 | 0.596 |  | 0.257 | 0.286 | 0.242 | 0.222 |
| 75/64 I am too shy or timid | 0.274 | 0.274 | 0.274 | 0.274 |  | 0.572 | 0.366 | 0.337 | 0.358 |  | 0.317 | 0.251 | 0.216 | 0.233 |
| 111/91 I keep from getting involved with others | 0.237 | 0.237 | 0.237 | 0.237 |  | 0.295 | 0.146 | 0.168 | 0.202 |  | 0.178 | 0.101 | 0.110 | 0.124 |
| **Partial Scalar Invariance Model** |  |  |  |  |  |  |  |  |  |  |  |  |  |  |
| 42/36 I would rather be alone than with others | 0.319 | 0.319 | 0.319 | 0.319 |  | 0.420 | 0.420 | **0.484** | **0.600** |  | 0.202 | 0.193 | 0.193 | 0.233 |
| 65/55 I refuse to talk | 0.167 | 0.167 | 0.167 | 0.167 |  | 0.139 | 0.139 | 0.139 | 0.139 |  | 0.107 | 0.092 | 0.083 | 0.101 |
| 69/59 I am secretive or keep things to myself | 0.439 | 0.439 | 0.439 | 0.439 |  | 0.620 | 0.620 | 0.620 | 0.620 |  | 0.257 | 0.287 | 0.242 | 0.223 |
| 75/64 I am too shy or timid | 0.273 | 0.273 | 0.273 | 0.273 |  | **0.568** | 0.385 | 0.385 | 0.385 |  | 0.317 | 0.251 | 0.216 | 0.233 |
| 111/91 I keep from getting involved with others | 0.238 | 0.238 | 0.238 | 0.238 |  | **0.295** | 0.195 | 0.195 | 0.195 |  | 0.178 | 0.101 | 0.110 | 0.124 |

*Note*. Non-invariant parameters are indicated in boldface.
